# Supplementary material for: Anti-Inflammatory and Pro-Regenerative Effects of Hyaluronan-Chitlac Mixture in Human Dermal Fibroblasts: A Skin Ageing Perspective
Source: Polymers (Basel). 2022 Apr 29;14(9):1817. doi: 10.3390/polym14091817 (PMC9105413; doi:10.3390/polym14091817)
Supplement: Supplementary file 1 [file polymers-14-01817-s001.zip › polymers-1684636-supplementary.pdf]

Supplementary file

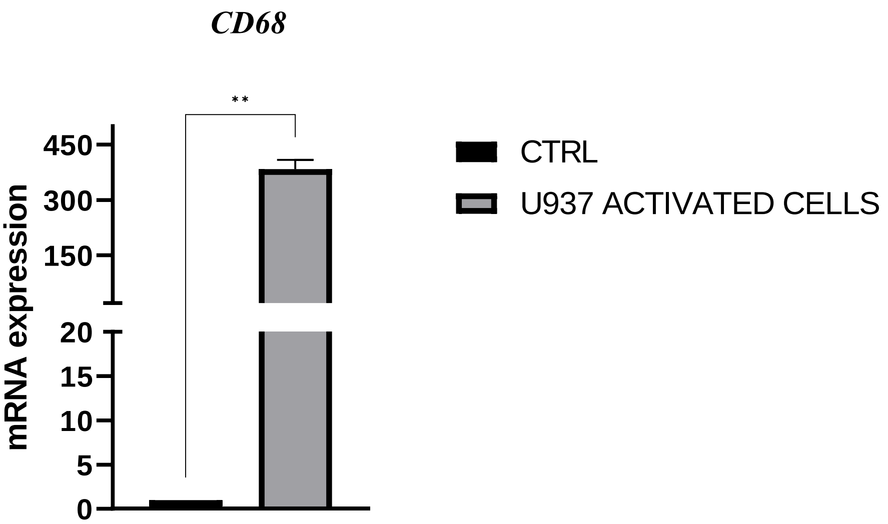

Figure S1. Detection of CD68 in activated U937 human monocytes. Cells were subsequently treated with PMA for 48 h and LPS for 1 h and then mRNA expression of the macrophage differentiation marker CD68 was evaluated by qPCR analysis after 24h. Data are expressed as mean ± SE of three independent experiments. Statistical differences are based on unpaired Student's t-test. **$P<0.01$ vs. untreated cells.

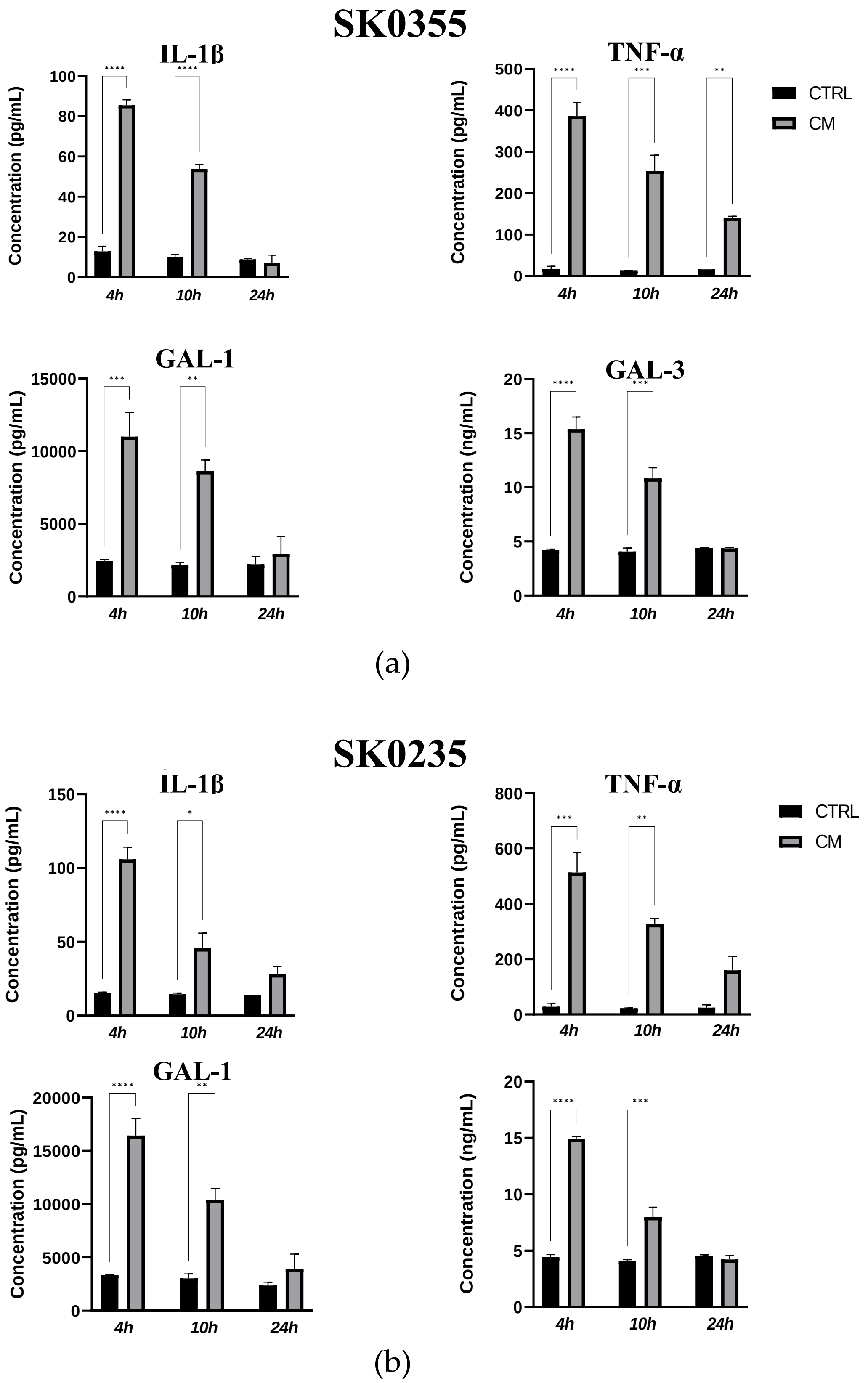

Figure S2. The conditioned medium (CM) of activated U937 cells induced pro-inflammatory molecules expression. Human dermal fibroblasts SK0355 (from 30-year-old woman, (a)) and SK0235 (from 54-year-old woman, (b)) were exposed for 24h to CM of activated U937 and then cultured under optimal conditions. Protein expression was evaluated by ELISA assays as described in the Materials and Methods section. Data are expressed as mean ± SE obtained from three independent experiments. CM, cell cultures exposed to U937 CM. *P<0.05, **P<0.01, ***P<0.001 and ****P<0.0001 vs untreated cells.
